# Robust Eye Center Localization through Face Alignment and Invariant Isocentric Patterns

**DOI:** 10.1371/journal.pone.0139098

**Published:** 2015-10-01

**Authors:** Zhiyong Pang, Chuansheng Wei, Dongdong Teng, Dihu Chen, Hongzhou Tan

**Affiliations:** 1 School of Physics and Engineering, Sun Yat-Sen University, Guangzhou, China; 2 School of Information Science and Technology, Sun Yat-Sen University, Guangzhou, China; 3 SYSU-CMU Shunde International Joint Research Institute, Foshan, China; Save Sight Institute, AUSTRALIA

## Abstract

The localization of eye centers is a very useful cue for numerous applications like face recognition, facial expression recognition, and the early screening of neurological pathologies. Several methods relying on available light for accurate eye-center localization have been exploited. However, despite the considerable improvements that eye-center localization systems have undergone in recent years, only few of these developments deal with the challenges posed by the profile (non-frontal face). In this paper, we first use the explicit shape regression method to obtain the rough location of the eye centers. Because this method extracts global information from the human face, it is robust against any changes in the eye region. We exploit this robustness and utilize it as a constraint. To locate the eye centers accurately, we employ isophote curvature features, the accuracy of which has been demonstrated in a previous study. By applying these features, we obtain a series of eye-center locations which are candidates for the actual position of the eye-center. Among these locations, the estimated locations which minimize the reconstruction error between the two methods mentioned above are taken as the closest approximation for the eye centers locations. Therefore, we combine explicit shape regression and isophote curvature feature analysis to achieve robustness and accuracy, respectively. In practical experiments, we use BioID and FERET datasets to test our approach to obtaining an accurate eye-center location while retaining robustness against changes in scale and pose. In addition, we apply our method to non-frontal faces to test its robustness and accuracy, which are essential in gaze estimation but have seldom been mentioned in previous works. Through extensive experimentation, we show that the proposed method can achieve a significant improvement in accuracy and robustness over state-of-the-art techniques, with our method ranking second in terms of accuracy. According to our implementation on a PC with a Xeon 2.5Ghz CPU, the frame rate of the eye tracking process can achieve 38 Hz.

## Introduction

Human eye localization plays an important role in estimating the focus location, as regards human computer interaction (HCI). The automatic detection and tracking of human eyes and, in particular, the accurate localization of eye centers, has been shown to be of considerable importance in many applications, such as advanced interfaces for biometrics, human-computer interaction, remote control, car driver surveillance, gaze direction, human attention control, the early screening of neurological pathologies, and facial expression recognition [1–3]. The accuracy and robustness of iris-center (IC) localization significantly affects gaze tracking performance. Eye localization systems can be broadly classified into two categories: active eye-localization systems (AELS) and passive eye localization systems (PELS). Recently, the AELS have improved significantly, and are beginning to play an important role in the HCI field. However, a typical AELS is generally expensive since it often requires auxiliary equipment (such as an infrared (IR)) camera [4] or a head-mounted device. Unlike the AELS, which uses the image coordinates of the pupil and corneal reflection [5–9], the PELS attempts to directly obtain information about the IC location based only on the images supplied from a camera video stream only [10–14]. Fortunately, further advancements have been made in recent years. Due to the increased speed of computer processors, improved computer vision techniques, and the advent of high performance digital cameras, the most desired type of IC localization output, which estimates the (x,y) coordinates of the user’s gaze (i.e., it directly maps the IC location to a target plane such as the monitor screen), can be easily implemented. Therefore, in this study, we investigate appearance-based IC locators, specifically focusing on those that can operate when only common digital cameras are available. Furthermore, we discuss approaches to improve the robustness and accuracy of this technology in detail.

Despite active research and significant progress in the last three decades, eye detection and tracking remains a very challenging task owing to the individuality of human eyes, occlusion of the eye by the eyelids, variability in scale, location, reflectivity, or head pose, eye open/closed cases, light conditions, etc. Various IC localization methods have been proposed in the literature, and can be roughly divided into three classes: (1) feature-based methods, (2) model-based methods, and (3) hybrid methods.

Feature-based methods use pertinent eye properties to detect candidate eye centers. The pertinent features may be edges, eye corners, or points, which are selected based on specific filter responses. The method proposed by Wang et al. [13] exploit the fact that the outer boundary of the iris is a circle, while Zhang et al. [14] describe an effective technique for estimating the gaze direction from the elliptical features [15] of one iris. Asadifard and Shanbezadeh [16] proposed an adaptive method based on the cumulative density function (CDF), which filters the eye image to determine the pixel values that are likely to belong to the pupil. In [17], the eye’s features, including the pupil center and radius, eye corners, and eyelid contours, are detected from frontal color facial images by integrating color information, Gabor features, and the mutual localization relationship between different features. Valenti and Gevers [18] propose an alternative voting scheme that uses isophote properties (i.e., curves connecting points of equal intensity) in the intensity image to detect the location of the eyes. However, since this method relies on maxima in the feature space, it may detect the eyebrow or eye corners instead of the IC when the number of features in the eye region is insufficient [19].The advantages of the feature-based methods are that they are robust to shape and scale changes and have lower computational complexity, without requiring any learning or model fitting. When the extracted features are not confused by noise or surrounding features, the resultant eye location can be very accurate. Nevertheless, the limitations of these methods are revealed in certain difficult scenarios, for example, when the user rotates his/her eyeball to either corner, when the user almost closes his/her eyes, or when there is occlusion due to eyelids or eye corners. The detected features might often be incorrect and, therefore, these methods frequently fail to accurately estimate the eye centers.

Model-based methods employ a prior model of eye holistic appearance and surrounding structures (or even the face) and often use classification of a set of features or the fitting of a learned model to estimate the location of the eyes. Moriyama et al. [20] use the generative eye region model, which can distinguish eye components by parameterizing the fine structure and motion of the eye. Hamouz et al. [21] proposed a method that search for ten feature points on the face using Gabor filters, apply a triplet of such corresponding features to define an affine transformation, provide a face hypothesis and, finally, verify the remaining configurations using two cascaded Support Vector Machine (SVM) classifiers. Kim et al. [22] propose a multi-scale approach to localize the eyes based on Gabor feature vectors, which is more robust with respect to initial points, while Niu et al. [23] introduce a two-direction cascaded AdaBoost framework for eye localization. The method by Realeet et al. [24] create a 3-D iris disk by mapping both the iris center and iris contour points to the eyeball sphere; then, a circle is fit to the iris to find the optimal eye ball rotation. Wang et al. proposed a method that[25] first applies statistically learned non-parametric discriminant features to characterize eye patterns, then determines probabilistic classifiers to separate eye and non-eye features and, finally, multiple classifiers are combined in AdaBoost to form a robust and accurate eye detector. By using both the global facial appearance and computer learning, model-based methods have the advantage of being very robust and well-suited for precise detection of overall eye location. However, the model-based methods have a drawback in that they usually require a large amount of training data to be collected and the model parameters need to be iteratively or manually adjusted; therefore, model-based methods are inappropriate for practical applications. Moreover, these methods usually fail to locate the eye centers when they are faced with subtle eye-center movements.

Hybrid methods aimed at combining the advantages of both feature-based and model-based methods within a single system have been developed to overcome the respective shortcomings of each approach. Huang and Wechsler [26] suggest an adaptive hybrid eye localization approach, using a consensus between navigation routines encoded as finite-state automata that explore the facial landscape to derive a saliency map, which is developed using genetic algorithms. These salient regions are then classified as eyes using genetically evolved decision trees. Wang et al. [27] treat faces as an image topographic manifold and use a terrain-classification procedure on the topographic manifold to generate a terrain map. In order to select proper eye pairs from the topographic manifold candidates, a SVM based on the Bhattacharyya kernel is applied. Valenti and Gevers [28] propose a hybrid scheme that uses isophote properties in the intensity image to detect eye location, and utilize mean shift and machine learning to overcome problems that arise in certain lighting conditions of due to occlusions from the eyelids.

Among all the methods, Valenti and Gevers [18], [28] achieved accuracy and efficiency. But this method may locate the eye center at eyebrow or eye eyelids which is far from the ground-truth position in some cases. To overcome the problem, in this paper, we apply face alignment model as a constraint to improve the robustness of eye center localization algorithm. We select the explicit shape regression method proposed in [29] to estimate the rough location of these points. This method uses regression ferns to build a two-level cascade which costs less in terms of computation and memory and achieves high accuracy. Since the estimation from the global shape model is not sufficiently accurate for a precise gaze-estimation system, we apply an unsupervised method to accurately locate the eye centers within the constraint of the global shape model. Based on robustness and accuracy considerations, we chose the method proposed in [18]. As an improvement, and to make use of our constraint, we select several estimated locations as our candidates. Subsequently, in order to reduce the magnitude of the calculation, we take the estimation which has the closest fit to the shape model as yielding the most likely eye–center locations. To match the estimation from unsupervised method and face alignment, we adopt an affine-invariant shape constraint (AISC) originally proposed in [30]. The experimental results demonstrate that the proposed method can locate the IC precisely and robustly, especially for profile views (face deflection angle greater than 30 degrees). The contributions of this paper are the following:

1. To solve the scales problem in eye center localization, several candidates of eye center location are selected in a scale space framework.
2. The face alignment and isophote curvature features are used together in an eye center localization system, which achieves a significant improvement in accuracy and robustness over state-of-the-art techniques.
3. To the best of our knowledge, we first demonstrate a robust eye center localization system on the non-frontal faces in FERET datasets.

The remainder of the paper is organized as follows: Section 2 presents our proposed eye localization algorithm, experimental results are given in Section 3 and, finally, the discussion and concluding remarks are given in Sections 4 and 5, respectively.

## Proposed Approach

**Fig 1:**
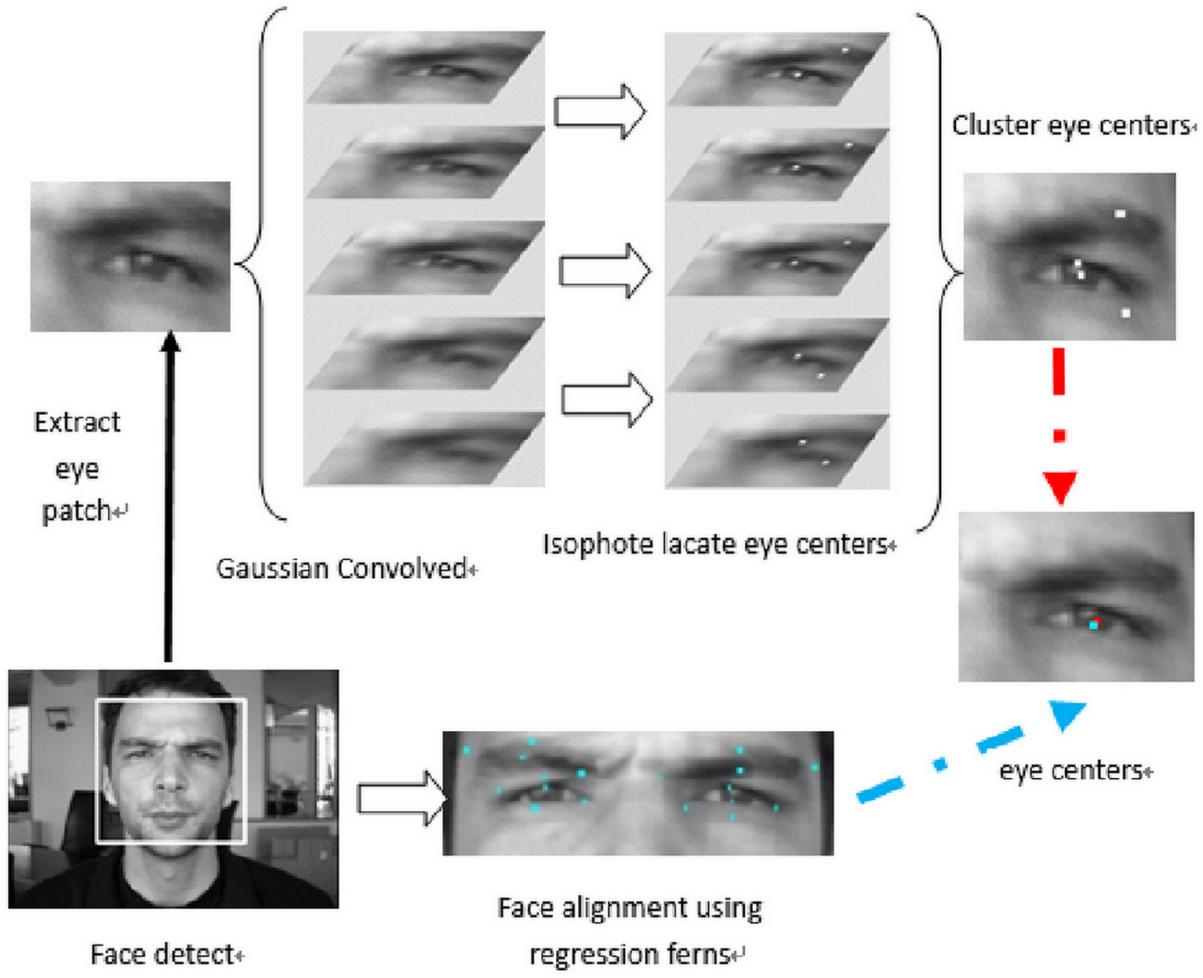
Flowchart of proposed eye-center location method. First, a facial detection process is applied, and then a series of facial landmarks are initiated using a facial bounding box. Regression ferns are applied to achieve facial alignment and the eye center location is estimated. A number of positions are selected as potential eye-center location candidates. Using the facial alignment results, the most likely eye centers are then determined.

In this section, we describe the proposed eye center localization system. A flowchart of the algorithmic procedures is shown in Fig 1. A face detection process [31] is first applied to the test image. With the bounding box obtained through the face detection, we initialize a series of facial landmarks. Next, we apply regression ferns to obtain a face alignment, a method which was described in [29]. Since this method applies the global information of human faces, it is more robust than the unsupervised method which only analyzes the information in eyes region. We take this estimation as a constraint to select the most likely eye center location in the next step. Meanwhile, we estimate the eye-center location using the method described in [18]. This method applies the isocentric patterns to estimate the eye center. To improve its estimation, different from [18] and [28], we generate Gaussian pyramid from the test image and select eye center locations in every patches of Gaussian pyramid as estimated eye-center candidates. The candidates of eye center locations reflect the estimation at different scales which achieve scale invariance in our method. Finally, combining results from both methods, we adopt the AISC [30] to reconstruct the facial landmarks and the candidate which minimizes the reconstruction error is taken as the eye center. This method has two advantages: (1) based on AISC, a series of landmarks estimated from the face alignments are used to codetermine the location which is more robust, (2) the eye center is selected from the candidates, which means we take the face alignment as a constraint and retain the accuracy achieved by unsupervised methods. The following subsections will explain the implementation specifics of each of the algorithmic procedures involved in this process in detail.

### Isophote eye-center Localization

It is well known that a significant feature of both the iris and pupil is their shape, which is considered to be circular in many cases. Specifically, the eye centers should maintain circular edges and, along these edges, the intensity of each pixel should be approximately the same. Given such prior knowledge, Valenti [18] [28] effectively estimated the eye centers using the maximum isocenter (MIC) based on isophote curvature information (the MIC method). This approach utilizes the isophote features through an unsupervised method which has shown accuracy as regards eye center detection. This method has three advantages: 1) low-computational cost; 2) robustness to changes in illumination, head pose, scale, occlusion, and eye rotation; and 3) suitability for low-resolution images. Isophote properties have been widely used in object detection and image segmentation [32–34].

Following this approach to representing the eye-center features, we use this method to obtain candidates for our technique. When it comes to eye-center localization, we can take the shapes of the pupil and iris as being the isophote curves. Hence, the eye-center location should be surrounded by these isophote curves and located at their center. To estimate the eye center, we should firstly find the isophote curves and then determine the radius, *r*. Therefore, we apply a Sobel operation to obtain the edges of the images and the curvature, *k*, of an isophote, which is the reciprocal of the subtended radius, and can be computed as

k=1r=−Ly2Lxx−2LxLxyLy+Lx2Lyy(Ly2+Lx2)32 (1)

Where *L*_*x*_, *L*_*y*_, and *L*_*xx*_, *L*_*xy*_, *L*_*yy*_ are the first- and second-order derivatives of the luminance function *L(x*,*y)* in the *x-* and *y-*dimensions respectively (for further details refer to [28]). Before we compute the derivatives of *L(x*,*y)*, we smooth the images using Gaussian filtering. Different Gaussian kernels can achieve different information depending on scale. We find that, in some cases, the choice of a Gaussian kernel is essential for eye-center estimation, as will be discussed below.

The orientation of *r* for each pixel can be computed by multiplying the gradient by the inverse of *k*. Combining *r* and the orientation of each pixel, we can estimate the displacement vectors of the estimated eye centers. The displacement vectors {*D*_*x*_, *D*_*y*_} to the estimated position of the centers are defined as

$$\left\{D_{x},D_{y}\right\}=-\frac{\left\{L_{x},L_{y}\right\}\left(L_{y}^{2}+L_{x}^{2}\right)}{L_{y}^{2}L_{xx}-2L_{x}L_{xy}L_{y}+L_{x}^{2}L_{yy}}$$ (2)

Taking these displacement vectors, every pixel in the image can be mapped into an accumulator (center map). Meanwhile, each pixel can be given a weighted curvedness to indicate the center location, such that

$$curvedness=\sqrt{L_{xx}^{2}+2{\text{L}}_{xy}^{2}L_{yy}^{2}}$$ (3)

**Fig 2:**
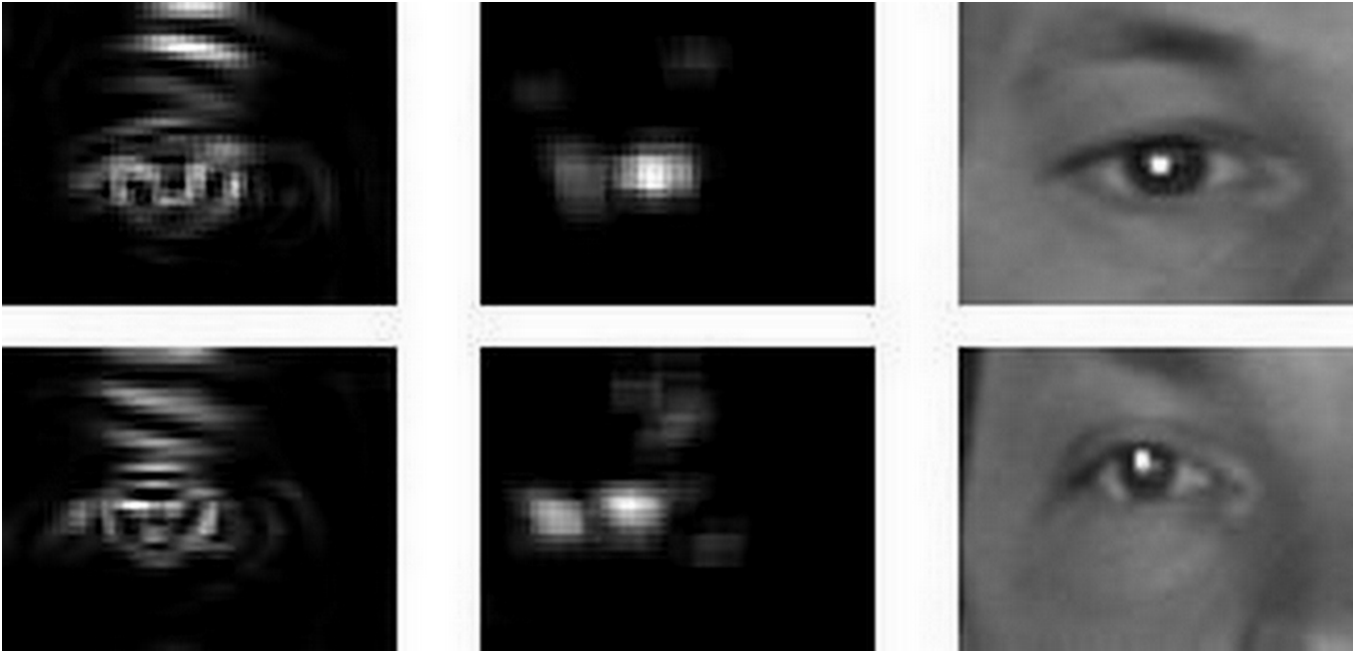
Determining the estimated eye-center location using the maximum isocenter (MIC) method. The left column shows the curvedness, the middle the center map, and the right the eye center estimation.

In the MIC method, the location that obtains the highest response on the center map is considered to be the estimated eye center location. Fig 2 shows this procedure.

### Multi-Scale Estimation

**Fig 3:**
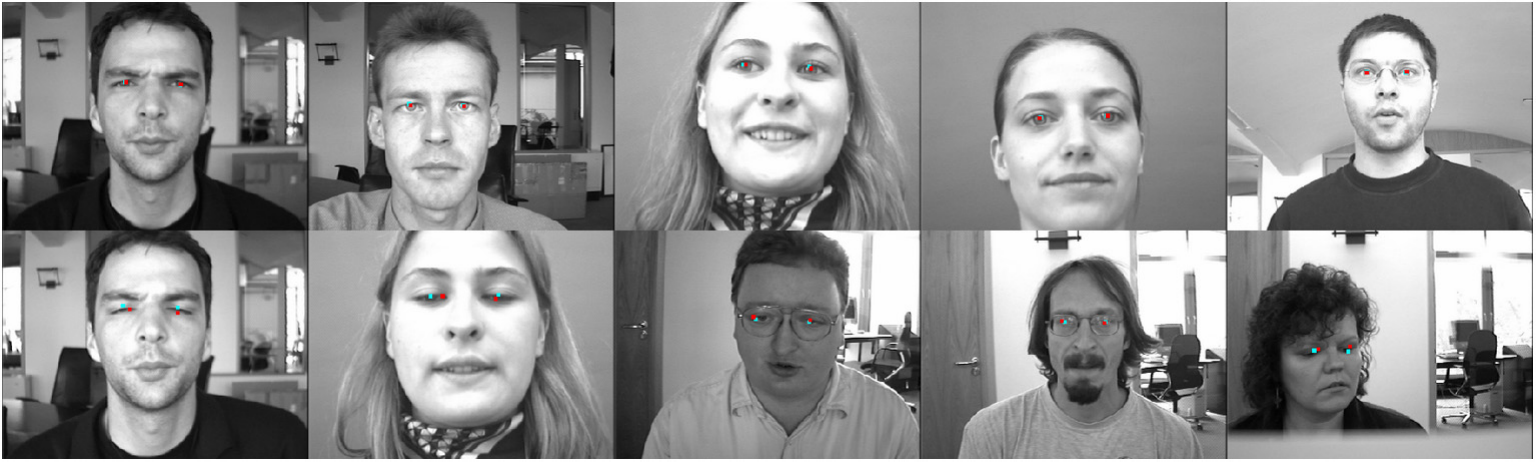
Selected BioID maximum isocenter (MIC) results. The first row shows correct results, where the real eye-center is identified, while the second row shows some typical errors due to non-ideal images.

In most cases, the MIC in the center map is the real eye center, as illustrated in the first row of Fig 3. However, in some non-ideal cases, the MIC may not represent the real eye center, as can be seen in the second row of Fig 3. Obtaining several candidate MICs from different center maps could be an effective strategy to overcoming these issues.

Therefore, along with the MIC method, Valenti extended his method using Scale Invariant Feature Transform (SIFT) and k-Nearest Neighbor (kNN) (MICs +SIFT + kNN) to select the most likely eye center location from the MICs. Furthermore, his later work [28], which applies this method to multi-scale image information, also achieves better results. This method can be considered to be an improvement on the MIC method or the MIC + SIFT + kNN method. Motivated by his work, we intend to estimate the eye center by using a different approach.

In detail, before we apply the MIC method, we first smooth the images using Gaussian filtering to eliminate the effect of noise. We found that differences in the Gaussian kernel sigma can significantly influence eye-center estimation accuracy. Hence, in our work, we use Gaussian kernels to generate the Gaussian pyramid, and obtain several center maps in different scales. Meanwhile, we not only select the MIC in the center map as our eye-center location candidate, but also the second largest points, and so on. Therefore, we actually select several candidate MICs for the real eye-center locations. When we select more and more MICs, it is more likely that at least one MIC point is the real eye-center location. However, if we select too many points from the feature maps, it becomes more difficult to determine which is sufficiently close to the actual eye-center location. Therefore, we generate five center maps using different Gaussian kernels and select the two biggest points from each center map.

### Face Alignment by Shape Regression

When the MICs have been selected from the multi-scale center maps, it is essential that a good classifier is built, in order to identify the most likely eye-center points. Previous works have attempted to use local information such as sift features to accomplish this. Although this approach has yielded better results [28], we wish to further improve the method performance by using global information from the whole face. This has the advantage that a face model built using global data is more robust, since the local features are not as stable as the global points in some scenes. Here, we use face alignment to locate semantic eye landmarks.

Most face alignment approaches can be classified into two categories: optimization-based and regression-based. Regression-based methods utilize a regression function that directly maps the image appearance to the target output, which is usually effective since the complex variations are based on sizeable training data and testing. For all regression-based methods, shape constraint is essential. Cao et al [29] proposed a novel regression-based approach without the use of any parametric shape models, which is called “explicit shape regression”. In our work, we apply the shape regression model to obtain the face alignment. We chose this model since it can be rapidly processed and achieves accurate results over a large range of pose variations.

Firstly, to illustrate the shape regression approach, we identify (*R* ^1^, *R* ^2^, …, *R* ^t^) as *t* weak regressors, and each *R* ^t^ contains *k* weak regressors (*r* ^1^, *r* ^2^, …, *r* ^k^). This is called two-level cascaded regression. During the training procedure, for every training sample, $\left\{\left({\hat{S}}_{i},I_{i}\right)\right\}$, *I*_*i*_ and ${\hat{S}}_{i}$ represent the image and real shape respectively, and each regressor aims to minimize the difference between the previous estimated shape, *S* ^*t*−1^, and the real shape, $\hat{S}$, with the following constraint:

$$R^{t}=\underset{R}{\text{arg min}}\;\sum_{i=1}^{N}\left\|{\hat{S}}_{i}-\left(S_{i}^{t-1}+R\left(I_{i},S_{i}^{t-1}\right)\right)\right\|$$ (4)

For each *R* ^t^, the input is the difference between *S* ^*t*−1^ and $\hat{S}$, and the output is the increment of shape represented by *δS*. Therefore, the regressor updates the shape each time the regression is applied, using features determined by the previous shape and input image, as expressed in the following equation:

$$S^{t}=S^{t-1}+R^{t}\;\left(I_{i},S_{i}^{t-1}\right)\;t=1,2,\cdots ,T$$ (5)

For the second level regressor, *r* ^*k*^, the features are determined by the output of *R* ^t-1^, but not *r* ^k-1^. Such a minute difference allows the features to be selected in a stable manner. A difference exists between *r*_*k*_ and *R*_*t*_ in that the features of each *r*_*k*_ are determined by the previous shape updated by the former regressor *R*_*t-1*_, but not *r*_*k-1*_, since it is unstable. This allows the feature to change frequently.

Different from the fregression forest, we apply random ferns as our weak regressor, *r* ^*k*^. Each fern is a composition of F features and use thresholds to divide the feature space into bins. For each training sample,we use the random thresholds for comparison against the feature extracted from it in order to determine which bin it falls into. All training samples must fall into one of the bins. For every bin, the regression output, *δS*_*b*_, which is defined in Eq (6), aims to minimize the alignment errors of the training samples falling into the bin, *Ωb* (*b* is a shrinkage parameter that prevents overfitting).

$$\delta S_{b}=\underset{\delta S}{\text{arg min}}\;\sum_{i\in \Omega_{b}}\left\|S_{i}-({\hat{S}}_{i}+\delta S)\right\|$$ (6)

The solution of Eq (6) is the mean of the shape difference, with

$$S_{b}=\frac{1}{1+\beta /\left|\Omega_{b}\right|}\frac{\sum_{i\in \Omega_{b}}({\hat{S}}_{i}-S_{i})}{\left|\Omega_{b}\right|}$$ (7)

We choose the difference of two pixels as our features. During every *R* ^t^, we randomly sample *P* points from the image and calculate *P* ^2^ features. For efficient regression, we select *F* features from every image, using a correlation-based method (for more details, see [29]). In order to achieve feature invariance between different scales after the features have been applied, we re-index the features coordinates by the the current shape, *S* ^t^. During the training process, we augment the training data, and randomly sample another 19 training samples as the initial shape of one training sample. This is effective as it improves the robustness of the entire process.

**Fig 4:**
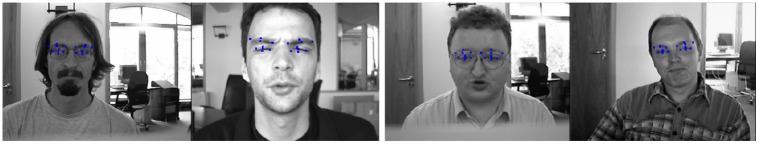
Facial alignment using random ferns. Explicit shape regression facial alignment approach applied to challenging eye detection cases. Occlusion due to various features and expression variations are examined. Reasonable detection results are obtained in some partially occluded cases (e.g., (a) and (c)), while accurate results are obtained for completely occluded cases, e.g., (b).

Fig 4 shows several challenging eye-detection cases, with examples of our eye detection results using the explicit shape regression face alignment approach. All these images contain expression variations and/or occlusions caused by hair, glasses, or pose. For some partially occluded components [such as the glasses in Fig 4A and 4C], our algorithm can give reasonable detection results. For those completely occluded components [such as the closed eyelids in Fig 4B], the results are accurate.

### Eye Center Location: Face Alignment and Isocentric Patterning

During testing, when the face detector successfully detects a face in the image, *I*_i_, we make use of the bounding box estimated by the face detector to initialize a shape, $S_{i}^{0}$. Then, we apply our cascade regression ferns {R^1^(r^1^, r^2^,…, r^k^), R^2^(r^1^, r^2^,…, r^k^),…, R^t^(r^1^, r^2^,…, r^k^)}, which contain *T* × *K* weak regressors, to estimate the. Meanwhile, we apply the multi-scale estimation which has been previously described to estimate the eye-center candidates, *C* (m_1_, m_2_,…) using isocentric positioning.

We adopt an affine-invariant shape constraint (AISC) originally proposed in [30] to select the most likely location of eye centers. Supposed that a face shape consists of *k* landmarks, the landmark of eye center can be reconstructed by the linear combination of its neighbors [30][35]. To apply the AISC, all the landmarks nearby could codetermines the position of eye center which make the estimation more robust.

In this paper, we select the landmarks around the eye centers and eye eyebrows as its neighbors, and apply the estimation by face alignment to reconstruct the eye center location, $m_{c}^{'}=M_{\omega_{c}}^{'}$, where *M* ^'^ denote k landmarks around eye centers, *ω*_*c*_ ∈ *R* ^*k*^ denotes the weights of the neighbor landmarks and *m* ^'^ denote the reconstruction of eye center. The point which is the nearest to the reconstruction of eye center (8) is taken as the final estimation, *m*_*est*_, with

$$m_{est}=\underset{m_{x}\in C}{\text{arg min}}\;\left\|m_{x}-m^{'}\right\|$$ (8)

## Experimental Procedure

### Database

We performed the experiments on the BioID [36] and FERET [37] databases, which were widely used in eye-center localization procedures in previous studies. The BioID database consists of 1,521 grayscale images of 23 different subjects that were taken in different locations and uncontrolled illumination. In the BioID images, some of the eyes are hidden by strong reflections on glasses, some are in closure, and some are turned away from the camera. For all these reasons, BioID is regarded as one of most challenging datasets in terms of eye-center localization. We selected all the samples in the BioID database to test our method.

The FERET database consists of 11,338 facial images of 994 subjects from different angles. Following the unsupervised eye-center localization method, we selected the *fa* (frontal face) datasets in FERET to test our technique. In addition, we also selected the FERET (non-frontal face) datasets for testing. All the faces in the datasets are turned left or turned right by specified angles, -22.5°, -15°, 15° and 22.5°, respectively.

Besides, in order to evaluate the effectiveness of our model for eye center tracking, we use the Talking Face Video (http://www-prima.inrialpes.fr/FGnet/data/01-TalkingFace/talking_face.html) to test the real-time performance of our algorithm. The Talking Face Video contains 5000 image consequences for a person engaged in a conversation.

### Parameter settings

The method proposed by Viola and Jones [31] is used to detect human faces. This method was also employed in OpenCV, and default parameters were chosen for the OpenCV face detector. Then, we used the mean facial shape given in training sets as the first initialized shape. By adding some perturbations, the remaining four initialized shapes were obtained. Following [29], we set *T* = 10, *K* = 500, *P* = 300, *F* = 5, and ᵦ = 1000. We used the LFPW database [38] including both training and test sets, with 29 annotated landmarks as training samples for the face regression model.

**Fig 5:**
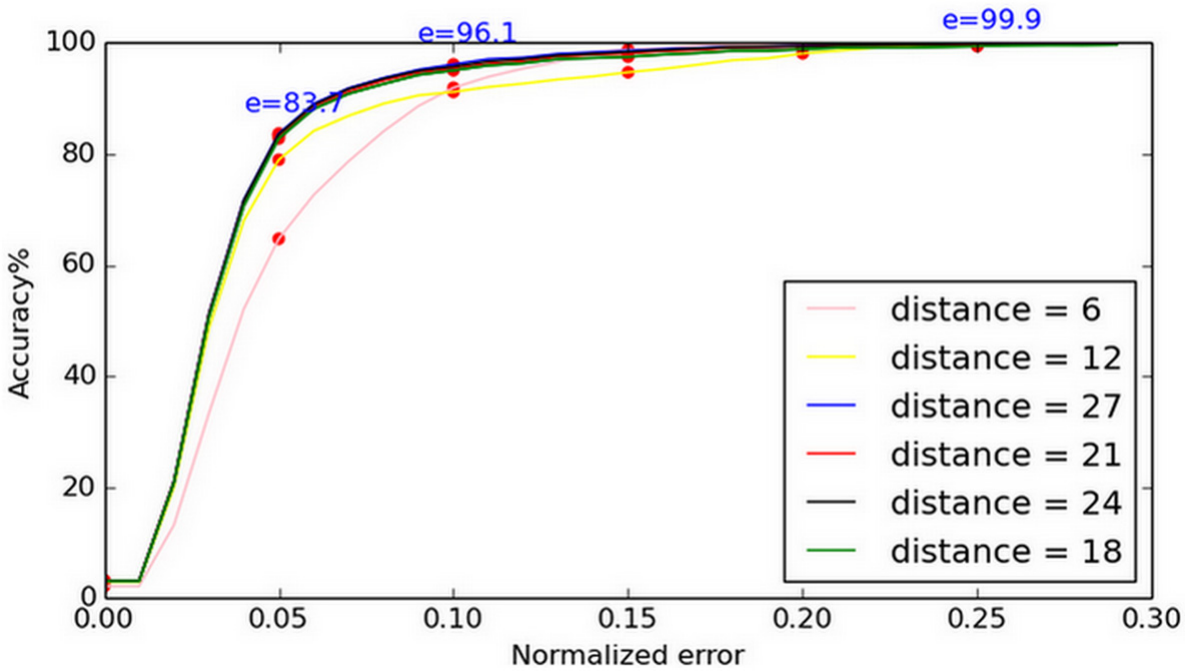
Accuracy achieved for varying distance. The distances between the chosen maximum isocenters (MICs) are varied, yielding noticeably different results.

When we apply the shape regression model, the initial shape is critical in many ways [29]. To achieve robustness, we initialized five shapes and used the mean result as the final estimation. For each center map, we selected two MICs. In general, the two largest points should be located close to the eye centers. However, it is pointless to select two close MICs as our candidates since this would be the same as selecting one MIC (because both points represent the same eye center). Instead, we are forced to choose the second largest MIC which is a given distance away from the first one. Depending on the specific distance we set, a different result is obtained, as illustrated in Fig 5 (tested on the BioID database). Based on our analysis, we chose the optimal distance (21 pixels) for estimation.

**Fig 6:**
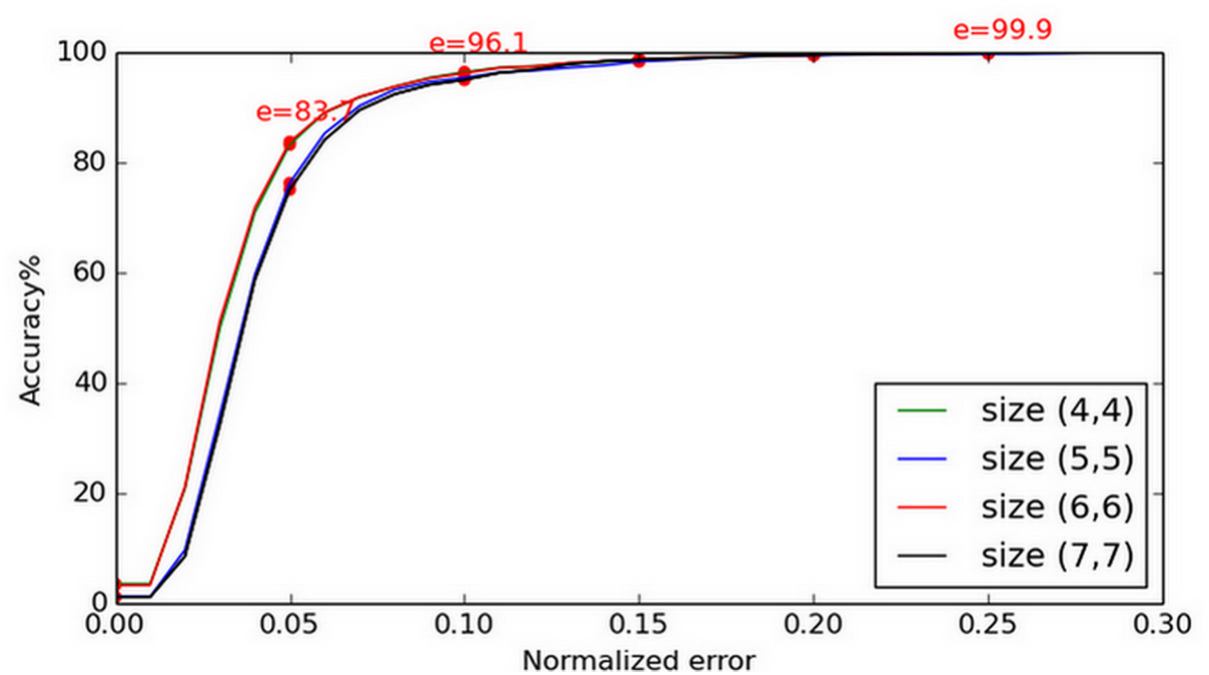
Accuracy achieved for varying kernel size. The center map is blurred before the maximum isocenters (MICs) are selected using a mean filter. The accuracy changes for varying kernel sizes.

Slight errors could cause inaccurate displacement vectors and, as a result, the distribution of the center map might not actually represent the most likely eye-center location. To achieve robustness, we blur the center map before we select the MICs. In comparison with the findings of [18] and [28], we found that a mean filter with size (6, 6) could achieve better results than a Gaussian kernel, although the optimal size should be determined by the image scale. After a number of experiments, we found this size to have good performance, if the scale was ignored. We illustrated our results using the BioID database in Fig 6.

### Procedure and Measurements

We adopted a normalized error to measure the eye-center localization accuracy. The normalized error was defined as the least accurate estimation of the two eye centers. The error can be expressed as

$$e=\frac{\text{max}(d_{left}+d_{right})}{\omega }$$ (9)

Where *ω* represents the distance between two real eye centers, and *d*_*left*_ and *d*_*right*_ represent the Euclidean distance between the estimated left and right eye-center positions and the real left and right eye-center locations, respectively. The normalized error was defined as the least accurate estimation of the two eye centers.

## Results

In order to investigate the subject performance of our face alignment method and the invariant isocentric pattern (FAIIP) eye-center localization approach, we compared our model with the original MIC method [18]. We chose to compare it with the original MIC because this technique has been extensively evaluated under different circumstances (e.g., low-resolution images, facial images with exaggerated expressions, illumination variation, pose, occlusion, eye rotation, resolution, and scale changes), and because it is directly related to our proposed method.

**Fig 7:**
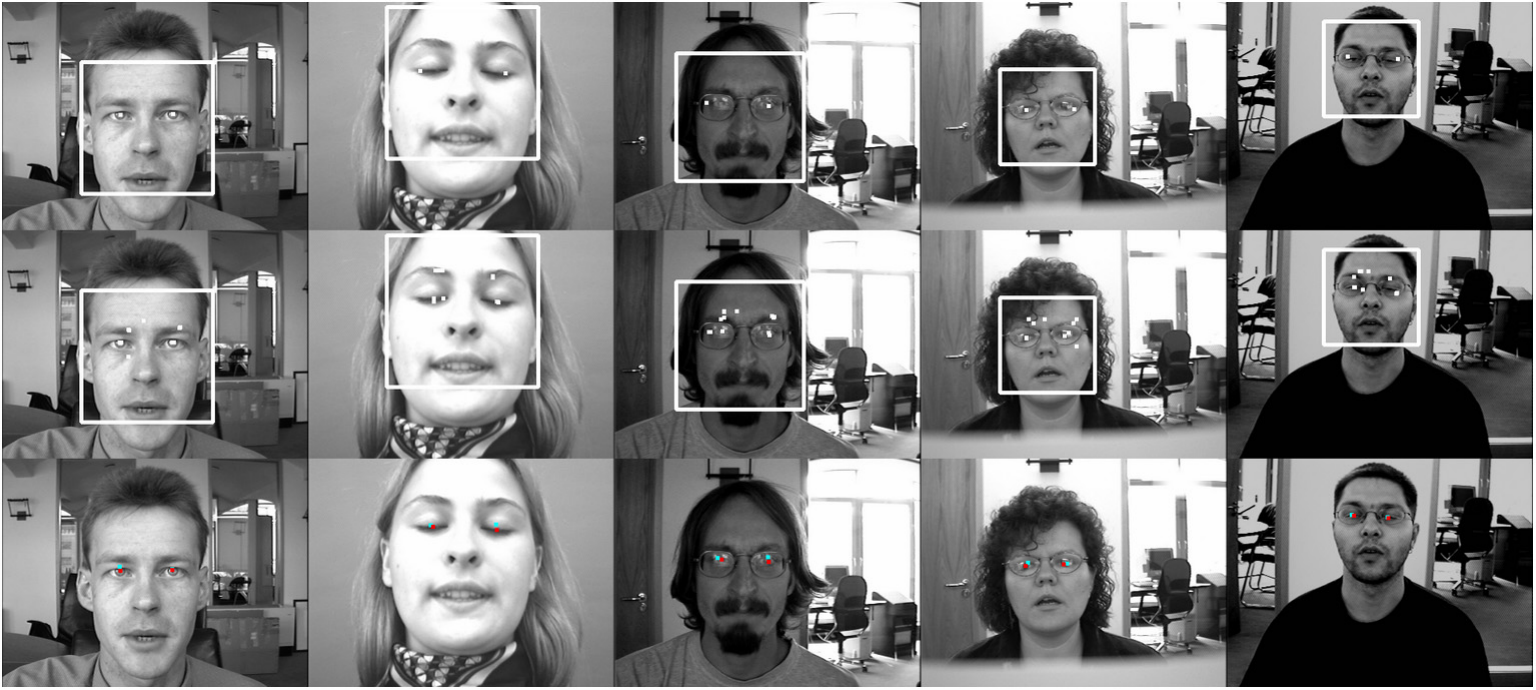
Successful (first row) and failed (second row) eye center localization using the BioID face database. The white dots represent the estimated centers and the first, second, and third rows shows the maximum isocenter (MIC) estimation results, the candidate MICs, and the final estimation (red dot) with the facial alignment constraint (blue dot), respectively. Both the present approach and the MIC method are effective in many situations (first row), however, in more challenging cases (other rows) our method has better accuracy.

In Fig 7, the first row shows the MIC estimation results, the second row demonstrates the candidate MICs, and the third row indicates the final estimation (red dot) with the face alignment constraint (blue dot). The first column shows that, in many cases, both our approach and the MIC method work well, while the other columns show that, in some extreme cases such as eye closure or strong reflection from glasses, our methods work more accurately.

**Fig 8:**
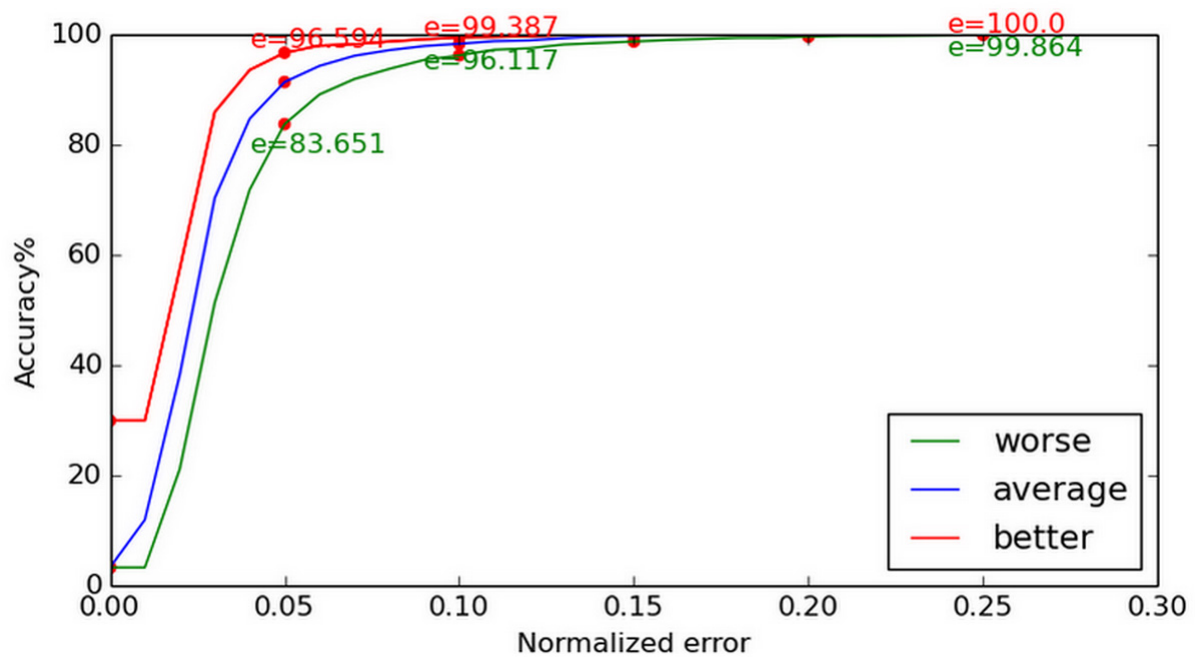
Normalized error curve on BioID. The optimal estimations, least accurate estimations, and the average difference between the two are given. Our method demonstrated better accuracy than the MIC method, when both were applied to the BioID database.

**Fig 9:**
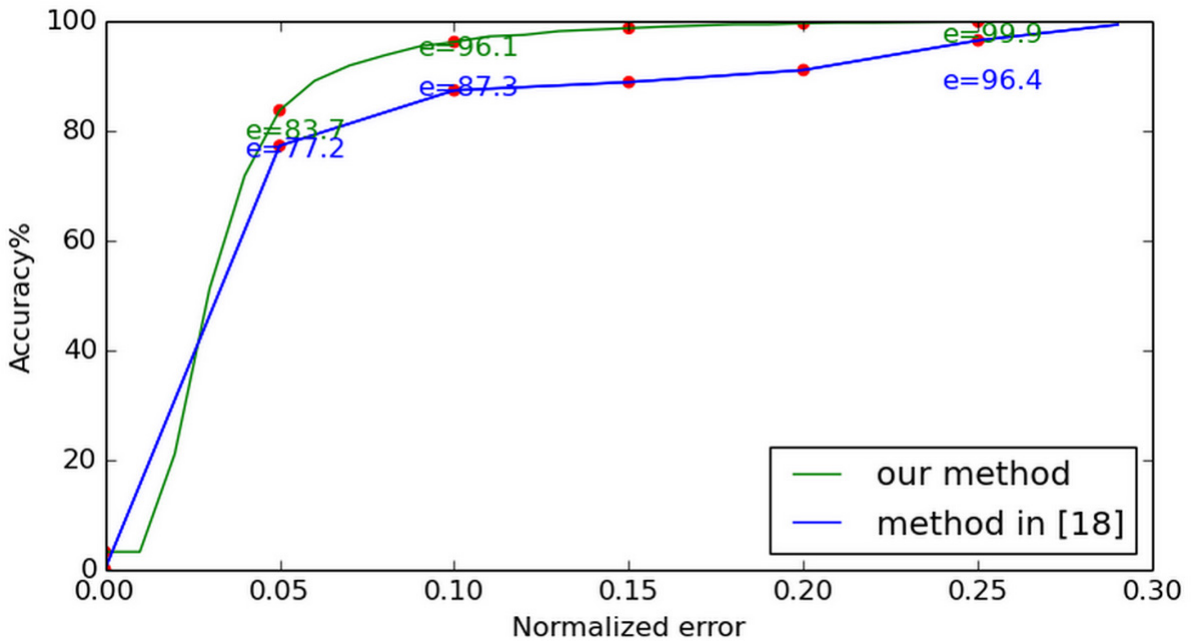
Comparison with [18] on BioID. The results yielded by our method when applied to the BioID and those given in [18] are compared. For (e ≤ 0.05) our method does not demonstrate significantly better performance, for (e ≤ 0.1) however, our method is more effective.

In Fig 8, apart from the least accurate estimations, we also show the optimal estimations and the average difference between these two results, which is required if our results are to be compared with other published works. Compared with the MIC method applied to the BioID database, our method yielded more accurate results, as shown in Fig 8. From the curve in Fig 9, we can determine that our method does not have a significant advantage at (e ≤ 0.05), as the face regression model does not work efficiently at this stage. But, when it comes to (e ≤ 0.1), our method is more effective, since the face regression model can work as a robust constraint to determine the most likely center location. When the normalized error is increased, the advantage of our approach is more significant.

Table 1 shows a comparison between our approach and the results of other works tested on the BioID database. At (e ≤ 0.05) and (e ≤ 0.1), our method’s performance is not significantly better. But, when it comes to (e ≤ 0.15), (e ≤ 0.2), and (e ≤ 0.25), our method achieves the most accurate results.

**Table 1:** Comparison of eye detection performance for different methods on the BioID database. The brackets indicate values that have been accurately measured from the respective authors’ graphs. Since some authors did not provide any graphical evaluation of the performance, e.g., by using a WEC curve, and the intermediate values could not be estimated, these missing values are denoted by “–”. (Partial data were obtained from [39], we will add the results of other works later).

Method	e ≤ 0.05	e ≤ 0.1	e ≤ 0.15	e ≤ 0.2	e ≤ 0.25
Leo [10]	80.7%	87.3%	88.8%	90.9%	(94.0%)
Markus [40]	**89.9%**	**97.1%**	–	–	99.7%
Timm [39]	82.5%	93.4%	95.2%	96.4%	98.0%
Asadifard [16]	47.0%	86.0%	89.0%	93.0%	96.0%
Niu et al [23]	(75.0%)	93.0%	(95.8%)	(96.4%)	(97.0%)
Valenti and Gevers [18]	81.9%	87.0%	(90.0%)	(97.0%)	98.0%
Valenti and Gevers [28]	86.1%	91.7%	(93.5%)	(97.1%)	97.9%
Campadelli [41]	62.0%	85.2%	87.6%	91.6%	96.1%
Cristinacce et al [42]	(57.0%)	96.0%	(96.5%)	(97.0%)	(97.1%)
Our method	83.6%	96.1%	**98.6%**	**99.4%**	**99.9%**

Since no methods exist which achieve the most accurate results for all e-values, we apply the ranking method of performance, which was first introduced in [39], to make a comparison between all the methods. In Table 2, we can clearly see that our method is more robust, as the normalized error is increased. In terms of the average rank, our score is second. Compared with the method given in [40], our method was less effective at (e ≤ 0.05) and (e ≤ 0.1). However, our result surpasses the accuracy of that method at (e ≤ 0.25). Since the method of [40] does not have results for the (e ≤ 0.15) and (e ≤ 0.20) regions (its error curves only illustrate the results achieved for (e ≤ 0.05) to (e ≤ 0.1)), we cannot conduct a full comparison. In addition, this model was trained using unpublicized data that was specially prepared for that study. Hence, it is difficult to replicate these results in different operating contexts.Also,the method in [40] was only tested on frontal faces. In contrast, we used images from the *fa* and *hl* datasets in FERET to show the efficacy of our model when applied to non-frontal faces.

**Table 2:** Rank at different normalized errors for data shown in Table 1. The last column shows the average rank of each method.

Method	e ≤ 0.05	e ≤ 0.1	e ≤ 0.15	e ≤ 0.2	e ≤ 0.25	Average rank
Leo [10]	6	6	8	8	10	7.6
Markus [40]	1	1	–	–	2	**1.33**
Timm [39]	4	3	4	5	3	3.8
Asadifard [16]	10	8	7	6	9	8
Niu et al. [23]	7	4	3	5	7	5.2
Valenti and Gevers [18]	5	7	6	3	3	4.8
Valenti et al. (kNN) [28]	2	5	5	2	5	3.8
Campadelli [41]	8	9	9	7	8	8.2
Cristinacce et al. [42]	9	3	2	3	6	4.6
Our method	3	2	1	1	1	1.6

**Fig 10:**
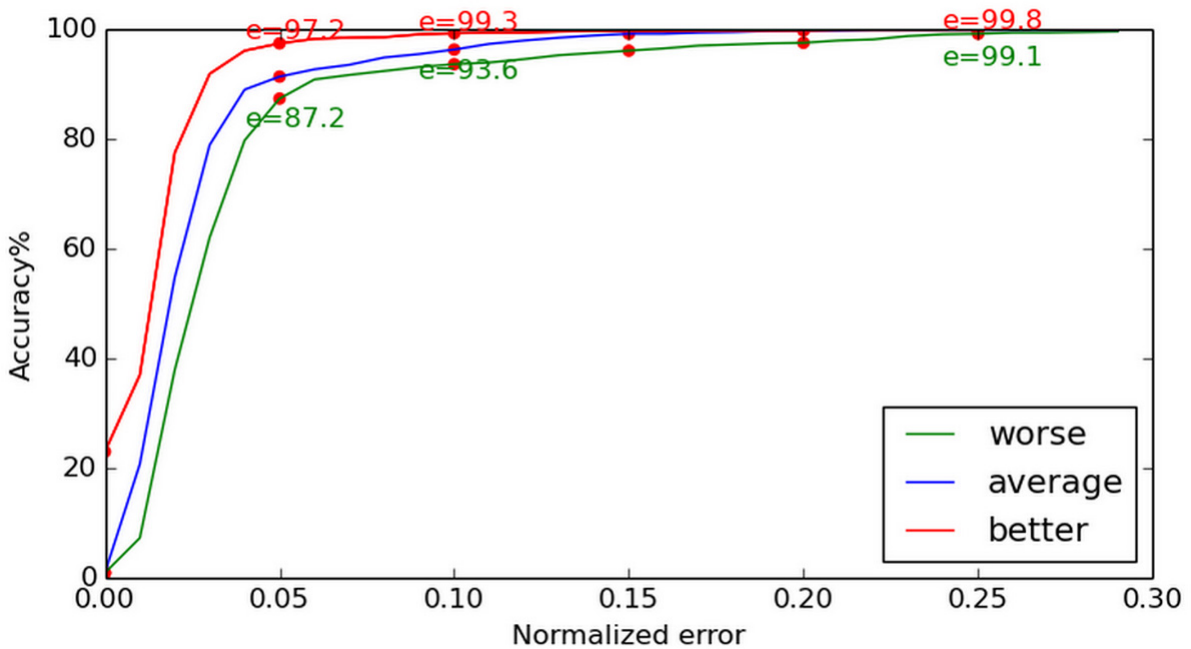
Normalized error curve on FERET. The most and least accurate estimations along with the average difference between them, for testing using the FERET database.

**Fig 11:**
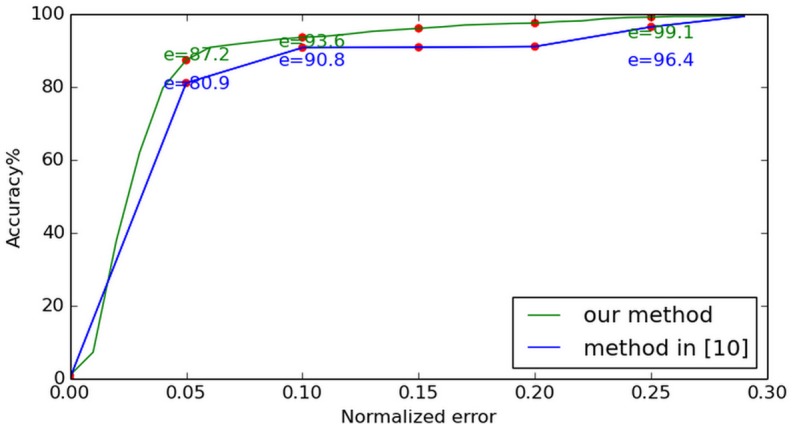
Comparison with [10] on FERET. The performance of our method is compared to that described in [10]. The method proposed here demonstrates better accuracy.

The same algorithm is also implemented on FERET database. Fig 10 shows the most and least accurate estimations and the average difference between them in FERET *fa* datasets. In [10], the authors proposed an unsupervised algorithm through differential geometry and local self-similarity matching to localize eye centers. Their experiments are also conducted on FERET *fa* datasets. Compared with the method described in [10] (see Fig 11), our method shows better performance. The least accurate estimation at (e ≤ 0.05) reaches 87.2% and, compared with the BioID result, the FERET results does not change significantly. This confirms our method’s stability.

Also, the non-frontal faces in FERET are selected to do a test. In [18] and [29], the previous work on the MIC method and face regression model show that they have both been applied to faces with different scales and poses. Hence, if we combine these two models, it is reasonable and acceptable that our approach can work well on non-frontal facial images. We select the faces turned left by angles of -22.5°, -15°, 15° and 22.5° to evaluate our method on non-frontal faces. In the Table 3, we list the amount of the images, the amount of the images which detect a face, and the accuracy (e ≤ 0.10) for each angle**.** For the non-frontal faces, the accuracy decreases, compared with the frontal faces. This can be attributed to three reasons: 1) the face detector is less stable, which leads to a worse initialization of facial landmarks. 2) Since the faces are turned left, the Euclidean distance between the two eye centered on the images must be smaller, as a result, the normalized error is bigger when the deviation is the same. 3) The eye center localization doesn’t work well.

**Table 3:** Accuracy on FERET non-frontal faces (e ≤ 0.1).

Angles	-22.5°	-15°	+15°	+22.5°
Images containing faces	760	609	320	760
Images detecting faces	706	606	305	630
Accuracy (%)	75.4	87.2	73.4	68.0

**Fig 12:**
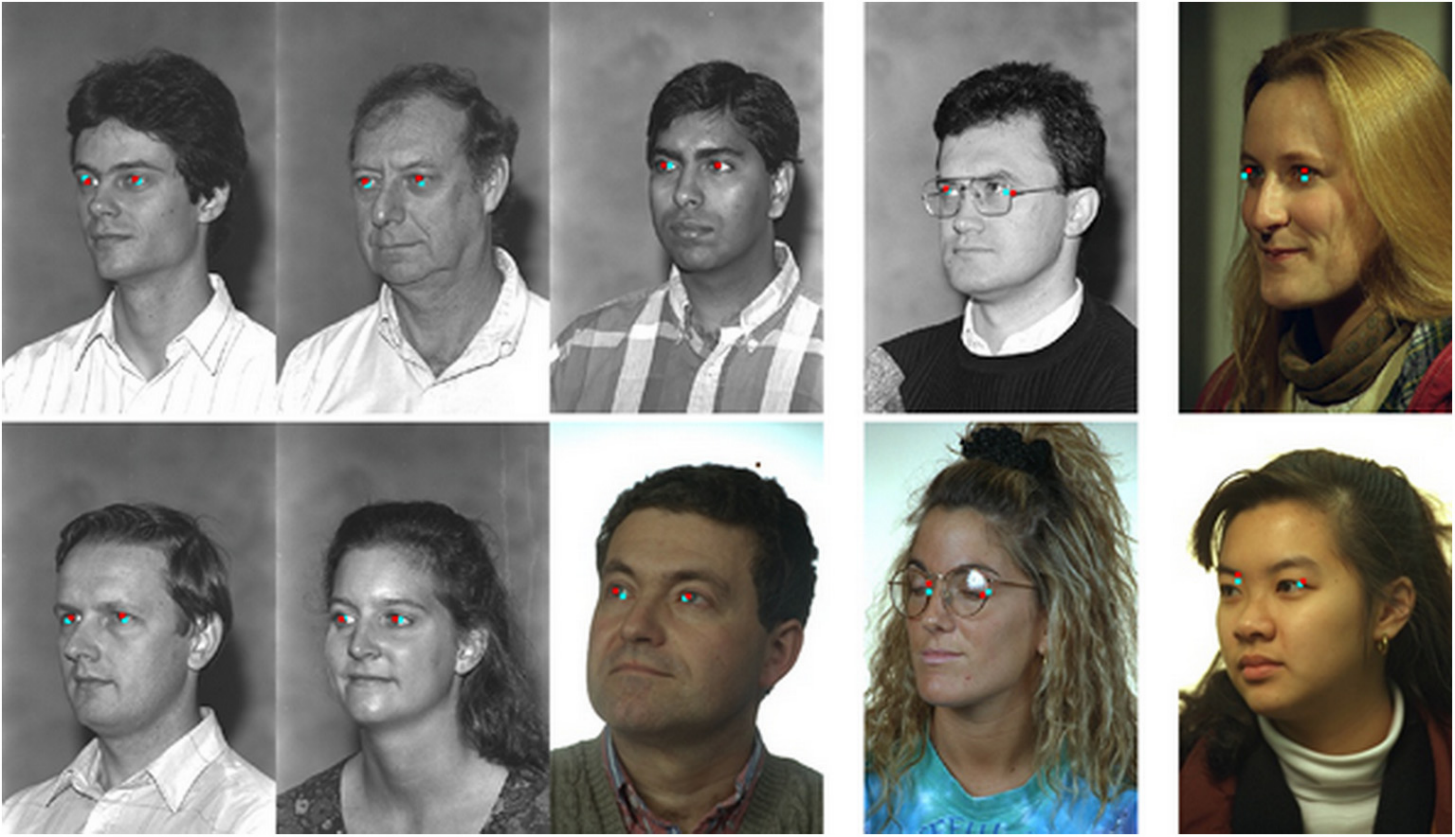
Examples of eye detection on the FERET database. The facial variations include pose, illumination, and glasses. The three left-most columns show accurate eye-center localization, while the right-most two columns show the inaccurate results.

In Fig 12, several examples show both accurate and inaccurate eye-center localization results. The first three columns show that, although the facial deflection angle is large, our algorithm works very well. In the fourth column, since the glasses generate a strong edge detection response when the face is turned left, these edges may lead to an inaccurate localization. In the fifth column, the facial angle is a little large, and the background causes instability in the eye-center estimation. Although the eye–center localizations are incorrect, the algorithm in this paper shows a very small deviation. This fully reflects the robustness of our method, due to the combination of global and local information.

In a real-time performance system, the image sequences are continuous. Therefore, a face detector is not necessary since the bounding box can be initialized from the estimated facial landmarks in the previous frame. Without the face detection, the processing time will be greatly reduced. The same method was also implemented in [18] and [40]. In our method, instead of applying the face detection operation, the estimated facial landmarks obtained from the previous frame are used to calculate a rough face bounding box. With this bounding box, the eye detection can be done following the algorithm proposed in the present work.

**Fig 13:**
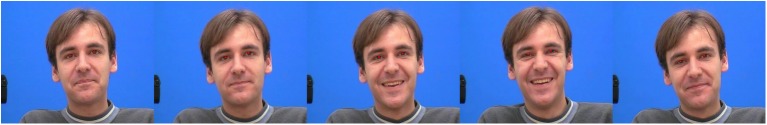
Sample results of eye tracking on Talking Face Video, selected from the 1th, 11th, 30th, 50th and 100th sequences in Talking Face Video.

The Talking Face Video database is used here to evaluate the developed tracking system. The video contains 5000 frames which are taken from a person engaged in a conversation. Explicit shape regression with five initialization shapes is used to do a face alignment. Python and C++ are taken as the programming language to implement the algorithm in a single thread based on the Xeon 2.5Ghz. The average processing time for each frame with a size of 720*576 is 26ms. Taking e≤ 0.10 as the successfully detecting eye centers, a 96.2% accuracy is got in 5000 images sequences. In Fig 13, some selected results are shown. The testing results are made into a video, which is uploaded online as: http://v.youku.com/v_show/id_XMTMwODY0Nzg4OA==.html (also available as supporting information S1 Video).

## Discussion

In our method, we first conduct a face alignment process, and then obtain the rough coordinates of both eye centers. This is robust but not sufficiently accurate, since the explicit shape regression method also takes advantage of global facial information, which is considered to be state-of-the-art. To achieve accurate eye-center localization, we use isophote curvature information to obtain several eye-center candidates. Among these candidates, we choose the one which is closest to the eye-center point (previously estimated by the shape regression model) as the accurate eye-center location.

When we apply this algorithm to the gaze estimation system, another advantage of our method can be found. As described in [43], the gaze estimation system needs both the eye-center and eye-corner locations to estimate the subject’s focus on the screen. Since we have obtained the eye-corner point during the face regression procedure, our algorithm significantly reduces the computation complexity of the gaze estimation system.

Although our method shows good performance and clear advantages during testing on the BioID face database, it is necessary to decide how many candidates should be estimated from each center voting feature map. A large amount of data is needed to modify the parameters. In our experiments, we select two candidates for both eyes in one center voting feature map and maintain five center voting feature maps using different Gaussian kernels from the input images. More candidates estimated from one center voting map may lead to a less accurate result, since the face regression shape model will have more choices and will find it more difficult to identify the most accurate location. Utilizing several candidates from different center voting maps has been shown to be effective due to clustering when the eyes are open, with no reflection from glasses and no occlusion. However, if the eyes are closed or the subject is wearing glasses with strong reflection, the standard deviation of the candidate’s coordinates increases. In some extreme cases, accurate eye-center locations could not be obtained for the candidates, which rendered our method unsuccessful. For the same reason, errors due to face shape may also cause uncertainty in our method.

To further improve the result, we believe that using a new measurement other than distance to select the candidate point with the best fit to the shape model would be effective. For example, since human eyes are asymmetric, locating the center based on another eye center point and conducting a cross-validation could be an effective approach in the future. Meanwhile, augmenting more training data that contains non-frontal faces for shape regression models would further advance the field.

## Conclusion

In this paper, by combining Valenti and Gevers' approach and the explicit shape regression method, we have proposed a more accurate method of eye-center localization. The explicit shape regression method gives a rough indication of the estimated eye-center locations but, while this method is robust, it is not sufficiently accurate. To achieve greater accuracy, we selected MICs from the eye regions by utilizing isophote curvature features. The strategy used to select candidates and to obtain the most likely eye centers was proposed in our paper. From the results, we found that our method is immune to some adverse conditions, because of the use of the global face shape model. In practical experiments, we illustrated the accuracy and robustness of our approach by comparison with previous methods. At the (e ≤ 0.15), (e ≤ 0.2), and (e ≤ 0.25) stages, we achieved accuracies of 98.6%, 99.4%, and 99.9%, respectively, on the BioID database. These results are more accurate than those presented in previous studies. By conducting a comparison of average results, we found that our method is ranked second amongst all the proposed techniques. However, compared with the most accurate approach, our method shows more accurate handling of non-frontal faces. We also conducted a test on the FERET *fa* and non-frontal faces datasets, and the accuracy of *fa* datasets at (e ≤ 0.05) was found to be 87.2%. Besides, tests for non-frontal faces and real-time performance system are also conducted. We discussed the gap between these results and concluded that our method works in approximately the same manner on both frontal faces and faces at an angle. From the results presented above, we conclude that our method obtains both robustness and accuracy in eye-center localization against changes in pose, scale, and other factors, by dynamically combining both global and local features. Finally, we present a real-time tracking system which can efficiently provide user’s eye positions in video.

## Supporting Information

S1 Video (MKV)
